# The roles of age at treatment and dose in carcinogenesis in C3Hf/Dp mice with a single administration of N-nitroso-N-methylurea.

**DOI:** 10.1038/bjc.1976.68

**Published:** 1976-04

**Authors:** B. Terracini, M. C. Testa, J. R. Cabral, L. Rossi

## Abstract

C3Hf/Dp mice were given a single i.p. injection of 50, 25 or 5 mug/g N-Nitroso-N-Methylurea (NMU) at either 1 or 70 days of age or 50 mug/g at 21 days of age. They were observed until death or until 120 weeks of age. The two highest doses of NMU produced tumours in a wide spectrum of organs, including the thymus, forestomach, lung, liver (only in males), kidneys, ovaries and orbital glands. The only two tumour types which appeared to be closely related to the occurrence of death were thymic lymphomata (most of which were found in mice dying before 40 weeks after treatment) and carcinomata of the forestomach. Lifetime analyses are presented concerning the occurrence of these two tumour types as well as the occurrence of any tumour after 40 weeks of age or since treatment. Incidences of thymic lymphomata were 67.6%, 39.0% and 21.2% in mice receiving 50 mug/g NMU at 1, 21 and 70 days respectively and 17.1% in mice receiving 25 mug/g at 1 day. In the other groups the incidence of thymic lymphomata was zero or negligible. The rate of progression of thymic lymphomata until death was related to both earliness of treatment and dose. On the contrary, incidences and progression of carcinomata of the forestomach were unrelated to age at treatment. Since breakdown of NMU is very rapid and does not require enzymes, these results are considered as evidence that host-tumour interaction differs from organ to organ. No excess of tumours over the controls was found in mice receiving 5 mug/g either at 1 or 70 days of age.


					
Br. J. Cancer (1976) 33, 427

THE ROLES OF AGE AT TREATMENT AND DOSE IN
CARCINOGENESIS IN C3Hf/Dp MICE WITH A SINGLE
ADMINISTRATION OF N-NITROSO-N-METHYLUREA

B. TERRACINI, MI. C. TESTA, J. R. CABRAL* AND L. ROSSIt

From the Division of Experimental Oncology A, Istituto -Nazionale per lo Studio e la Cura

dei Tumort, Via G. Venezian 1, 20133 Milan, Italy

Received 25 March 1975  Accepted 19 December 1975

Summary.-C3Hf/Dp;- mice were given a single i.p. injection of 50, 25 or 5 ,ug/g
N-Nitroso-N-Methylurea (NMU) at either I or 70 days of age or 50 flg/g at 21 days
of age. They were olbserved until death or until 120 weeks of age.

The two highest doses of NMU produced tumours in a wide spectrum of organs,
including the thymus, forestomach, lung, liver (only in males), kidneys, ovaries and
orbital glands.

The only two tumo'lr types which appeared to be closely related to the occurrence
of death were thymic lymphomata (most of which were- found in mice dying before
40 weeks after treatment) and carcinomata of the forestomach. Lifetime analyses
are presented concerning the occurrence of these two tumour types as well as the
occurrence of any tumour'after 40 weeks of age or since treatment.

Incidences of thymic lymphomata were 67^60%, 39-0? and 21-20% in mice receiving
50 ,ug/g NMU at 1, 21 and 70 days respectively and 17.1% in mice receiving 25 ug/g
at 1 day. In'the other groups the' incidence of thymic lymphomata was zero or
negligible. The rate of progression of thymic lymphomata until death was related
to both earliness of treatment and dose. On the contrary, incidences and progression
of carcinomata of the forestomach were unrelated to age at treatment. Since
breakdown of NMU is very rapid and does not require enzymes, these results are
considered as evidence that host-tumour interaction differs from organ to organ.

No excess of tumours over the controls was found in mice receiving 5 ,ug/g either
at 1 or 70 days of age.

THE CARCINOGENIdITY of N-nitroso-
N-methylurea (NMU) in different species
of laboratory animals i? well"estab-lished
(IARC, 1972). A single adminiAtration
of this chemical 'can induice'a high yield
of tumours at diff4rent sites in mice and
rats (Druckrey et al., 1964; :Graffi and
Hoffmann, 1966;- Terracini and Stramig-
noni, 1967; Kelly et al., 196'';' Leaver,
Swann and Magee, 1969-; Frei, 1970;
Joshi and   Frei, 1970; Terracini arid
Testa, 1970; Frei, 1971). NMU- is ' also
known to undergo rapid deconmposition
at physiological pH  (Druckrey et 'al.,
1967; Swann, 1968). Thus' a simple ex-
perimental system is `provided where

some biological aspects of carcinogenesis
(~ncidence,'target organs, progression and
latent period) can be investigated' by
changing one parameter of exposure such
as age.at treatment or dose.

The, present study reports a lifetime
analysis of the carcinogenicity of a single
admninistration of 5, 25, or 50 ,ag/g NMU
to C3Hf/Dp mice aged 1, 21 or 70 days.

MATERIALS AND METHODS

Inbred C3Hf/Dp mice from this laboratory
were used. They were maintained in air-
conditioned rooms at the temperature of
20-24?C, in plastic or bakelite cages and
given a commercial diet in pellet form

* Present address: Eppley Institute for Research in Cancer, University of Nebraska Medical College,
Omaha, Nebraska, U.S.A.

t Present address: Istituto di Oncologia, Universith, Genova, Italy.

B. TERRACINI, M. C. TESTA, J. R. CABRAL AND L. ROSSI

(Piccioni, Brescia) and tap water ad libitum.
Recrystallized NMU was obtained through
a kind gift from Dr P. F. Swann, Courtauld
Institute of Biochemistry, Middlesex Hospital
Medical School, London. NMU was dis-
solved 0.1%  in saline immediately before
use and injected once i.p. Eight groups
of mice were assembled, including the
controls receiving only saline (Group? I).
Groups II and III were given 25 ,ug/g NMU
at 1 and 70 days of age respectively. Groups
IV, V and VI received 50 ,tg/g NMU at
1, 21 and 70 days of age respectively. Two
additional groups were treated with 5 ,ug/g
NMU at 1 day (27yy and 17X) and at 70
days of age (19y? and 20,-3). Results for
these additional groups are not included in
the tables and figures as they did not differ
from those observed among controls.

Mice treated at 1 or 21 days of age were
left with their mothers until weaning, which
occurred at 25-28 days of age. At weaning,
a few animals from some groups were used
for purposes outside the present report.
Also 3 mice from the control group were
lost during the experiment. All other mice
were allowed to die naturally or were killed
with ether when moribund or if they survived
to 120 weeks of age. Mice were examined
daily and weighed at weekly intervals until
10 weeks of age and then at fortnightly
intervals.

All mice were submitted to autopsy
including opening of the skull but not of
the spinal cord. The thymus, liver, kidneys,
spleen and orbital glands as well as any
organs showing gross abnormalities were
fixed in Bouin's fluid and histologically
examined in sections stained with haema-
toxylin and eosin.

Cumulative probabilities of dying with
tumours at any site, thyhic lymphomata
or carcinomata of the forestomach have
been calculated according to the formula:

- 1 (Nf1-t1)
Pn-      N -    -. )

N1

(Nv2 - Y 2       (Nn -tn)

N2               Nn

where Pn represents the probability of
having died with that event by the end
of week n, N is the number of animals
alive at the beginning of each week and
t is the number of animals dying with that

event during each week (Saffiotti et al.,
1972). Since the value of P changes only
for weeks when one or more events are
recorded, only these weeks are plotted in
Fig. 2, 3, 4 and 5.

RESULTS

Pre-weaning mortality

Among control mice, 10/72 (13.9%)
died during the suckling period. Follow-
ing treatment with NMU at 1 or 21
days, the proportion of mice dying before
weaning ranged from 5/96 (5.2%) in
Group V (50 ,tg/g NMU at 21 days)
to 15/83 (181lOo) in Group IV (50 ,ug/g at
1 day). None of the differences between
treated groups and controls was signifi-
cant at the 0 05 level.

Weight gain

Body growth was depressed in both
sexes following 25 or 50 ,tg/g NMU at
1 or 21 days of age but not at 70 days
of age. Growth depression was obvious
before weaning and affected all the mice
whether they subsequently developed
lymphomata or not.

Mortality and evaluation of causes of death

The numbers of survivors at different
ages in both sexes of all groups are given
in Table I. Over 50%     weaned mice
were alive at 110 weeks of age in controls.
Survival was poorest in Group IV (50
,ug/g at 1 day) where all the mice died
before 73 weeks of age. In Groups II,
V and VI (25 ,tg/g at 1 day and 50 ,tg/g
at 21 and 70 days respectively) no sur-
vivors were left at 100 weeks of age.
In comparison with these, mice of Group
III (25 ,ug/g at 70 days) showed a better
survival rate, but their lifespan was still
shorter than that of the controls.

Distribution of deaths vs. time in
the whole population of mice under study
is represented in Fig. 1, which indicates
a bimodal pattern of mortality distribu-
tion. All 107 mice dying before 40 weeks
of age belonged to Groups II, IV, V or
VI and 89 of them had a thymic lymph-

428

AGE AND DOSE IN CARCINOGENESIS BY NMU

TABLE I.-Numbers of C3Hf/Dp Mice Surviving at Different Ages Following

NMU Injection at 1, 21 or 70 Days of Age

Dose     Age at      No. of
NMU     treatment     mice

Groups    (,ug/g)   (days)    weaned*

I     controls      1         25

c 34
II       25         1           25

< 16
III       25        70          20

3 20
IV        50         1          44

3 24
V        50        21          38

d 44
VI        50        70          41

& 30
* Or treated, at 70 days.

30 4

(o

-C

0) 25 -
o

4;  20 -

U

Z. 15

Co

E S

C)-

CD
.-

0
C.)

0) 1

0.

0)

Cl)

Survivors at weeks of age

Av                    --,

10
25
34
24
15
20
20
44
23
37
43
41
30

20
25
34
20
14
20
20
21
10
33
38
41
30

30
25
34
19
14
20
20
12
4
22
34
40
27

40
25
34
18
13
20
20
10
4
19
31
37
23

50
24
34
18
13
19
19

6
4
16
27
34
23

60
24
33
16
12
16
18

3
3
9
23
27
18

70
24
33
12

7
14
17

1
0
2
20
13
14

80
24
31

2
6
9
16

0

1

8
5
11

90
24
30

0
3
4
10

0
4
0
5

100
21
23

0

0

7

0

1

110
17
21

120
11
17

4   3
0

I   I            I        I        I       I        I       I        I        I       I   -  - T

0-9     10-19    20-29   30-39    40-49   50-59     60-69   70-79   80-89    90-99    100-109 110-119

- Age (weeks)

...... Weeks since treatment

FIG. 1.-Age-specific mortality rates. All groups including controls are pooled.

oma. Five of these mice also had 1-2
small lung adenomata, one had a papil-
loma of the forestomach and another
had a renal adenoma: none of these
tumours was likely to be causally related
to death. Tumours observed before 40
weeks of age in lymphoma-free mice
were 2 lung adenomata, 1 mammary
tumour, 1 adenocarcinoma of the intestine
and 1 carcinoma of the forestomach.

Twelve mice died with thymic lympho-
mata after 40 weeks of age, 7 of which

also had tumours at other sites (3 lung
adenomata, 2 papillomata and one car-
cinoma of the forestomach, 2 granulosa-
cell tumours of the ovary, one renal
adenoma and 1 subcutaneous sarcoma).
It was assumed that in these 7 mice
death had been caused by the thymic
lymphoma.

In lymphoma-free mice dying after
50 weeks of age, a frequent observation
at autopsy was multiple tumours, none
of which could be considered as the main

429

B. TERRACINI, M. C. TESTA, J. R. CABRAL AND L. ROSSI

cause of death with the possible exception
of mice with carcinoma of the fore-
stomach. These carcinomata replaced a,
large part of the stomach wall, invaded
the peritoneum and were complicated
by purulent inflammation: they could
therefore have had a major role in causing
death. In 6 animals the only tumour
present was carcinoma of the fore-
stomach.

Lifetime analyses were undertaken
considering the following end points: 1,
death with thymic lymphoma at any
age. 2, death with tumours at any site
in mice surviving at 39 weeks of age or
39 weeks after treatment (excluding mice
with thymic lymphomata). 3, death with
carcinoma of the forestomach. Inci -
dences of other tumours have been
evaluated considering the proportion of
mice developing each tumour type among
survivors at 40 weeks of age.

Distribution of tumours

For both sexes of each group Table II
reports the number of tumour-bearing
animals (TBA), mice dying with more
than one tumour and mice developing
tumours at specific sites. Only the in-
cidences of extrathymic lymphomata,
mammary and subcutaneous tumours
were not affected by treatment with
NMU. Considering males and females
together, in all treated groups the pro-
portion of TBA was over 8000 v 55.900
in controls. Mice dying with more than
one tumour were 18.60o in controls,
23-5%  in mice given 50 ,ug/g NMU at
birth (Group IV, with poor survival
rate) and ranged between 46 and 5900
in other groups. The average number
of tumours per mouse ranged between
0-83 in controls and 2 09 in Group VI.

No sex differences were observed
concerning the proportion of TBA and
that of mice dying with thymic lymphoma,
or carcinoma of the forestomach, i.e.
those parameters for which lifetime analy-
sis has been undertaken on both sexes
considered together. In fact, apart from

ovarian, mammary, uterine and vaginal
tumours, a sex difference in response was
detected only for liver-cell tumours. Con-
sidering the whole population under study
(including the 2 additional groups treated
with 5 ,ug/g NMU) these were found
in 15/239 females and 80/205 males
(P < 0 001).  The  difference between
15/239 females and 6/205 males developing
renal tumours is not significant at the
0 05 level.

Induction of thymic lyrniphomata

Overall incidences and results broken
down into 20-week periods after treatment
are reported in Table III. Thymic lym-
phomata were consistently observed in
all groups treated with 50 /Ig/g and in
mice receiving 25 ,ig/g at 1 day of age:
cumulative probabilities of dying with
thymic lymphoma in these groups are
represented in Fig. 2. Incidences in
mice receiving 50 lig/g NMU at 1, 21
or 70 days of age (Group IV, V and
VI) were 67.6%, 39-0 0    and  21.2%
respectively. Reducing the dose given
at 1 day to 25 /ig/g NMU (Group II)
produced a decrease in the incidence
of thymic lymphomata to 17-1 00. One
thymic lymphoma was found in one
mouse out of 40 given 25 uig/g at 70 days
of age and none in the controls or in
mice receiving 5 ,ig/g either at 1 or 70
days.

Age at treatment affected both the
incidence of thymic lymphomata and
the time elapsing between treatment
and death caused by a thynmic lymphoma.

During the first 20-week period after
treatment with 50 ,Ig/g NMU the pro-
portions of mice dying with thymic
lymphoma were 35/68, 12/82 and 4/71
respectively in Groups IV, V and VI
(the differences between Groups IV and
V and Groups IV and VI are significant
at the 0 001 level whereas that between
Groups V and VI is not significant at
the 0-05 level). Between 20 and 39
weeks after treatment, the proportions
of mice dying with thymic lymphoma

430

AGE AND DOSE IN CARCINOGENESIS BY NMU

n~~~~~

-4-5

0 ~S

r ~~~~~~~~~~~~~~~

-                    0i  - 1-!  ?  ?
C0I

>  *-~~~
0~~~~~

P-Q

*_ Z      0: 00 O 00 0 _ r0 0 _

o
" Q

-       ; t O  -   --  -4 -o

0.

X   C;.=;  o- < X o e X < o _ s _ 0 C  >
0~~~~~~~~~~~~~~~~~~~~~~0c 0

O q cz   N  N cq  cq m
CO~~~~~~0

o      n W0 S.5      0 o  <  o1  C0 r0

ti-4        -  -  0

431

.0

0

-"

*4 N
0

$0.4
~0

0
C'

0
0

-3

0
bf)

0 1

z

0

0._
-

o

"0

1-

>._
F._

4-

rn.
0
0
S

C)

0

0c
0
0
Cc
0
ea

1.4
0

5),
0'

I
II

B. TERRACINI, M. C. TESTA, J. R. CABRAL AND L. ROSSI

> c

4-1 ~ ~ ~ 0 0

I~  I ;;X t

-~~~~~~~~~~~0t

4L n 4 C   C

i  ?!h iI   r  4

cd

t~~~~~~~   4- IC r- r I  cl o  _  Xs

-E--
u  8-  0

CI r  0 r -   0   ] i-
Z        Cd;  r -   ? '; - ???

.~ ~ ~~~~~~L IM It  cc (X

_  *K,  ^  o0 a:bio U

t~~~~~~C  *S  IC  "It  ?z  00   X nn
t > C Q r.O =s o _ _ _4X

432

AGE AND DOSE IN CARCINOGENESIS BY NMU

4*

a.

a
a
S

i 0?
4        O

0

P-  M     r

.

S
S
S
S

o0
iOL  4

0
0
:)0

I          I         I         I
10        20        30         40

Age (weeks)

50        60       70

FIG. 2.-Probability of dying with a thymic lymphoma in C3Hf mice given a single injection of NMU.

v 25 pg/g at I day (Group II)
* 50 ,ug/g at 1 day (Group IV)

a 50 p1g/g at 21 days (Group V)

050 /ig/g at 70 days (Group VI)

were similar in Groups IV and V, i.e.
11/31 vs. 18/68 (P > 0-05) while it was
only 6/67 in mice treated at 70 days
(P < 0*05). Finally, from 40 weeks after
treatment, thymic lymphomata were
found in 0/14, 2/47 and 5/57 mice treated
at 1, 21 and 70 days respectively. The
difference regarding the risk of dying
with a thymic lymphoma related to the
earliness of treatment was therefore much
more obvious in the early than in the late
phases of the study.

In both Groups II and VI it appears
that the relatively low incidence of
thymic lymphomata was paralleled by a
delayed time of death.

The distribution of thymic lympho-
mata among the 13 litters given 50 ,ug/g
NMU at-birth and the 13 litters receiving
the same dose at 21 days was analysed
by comparing for each litter the actual
number of mice developing lymphomata
with the number expected assuming a
uniform distribution throughout all litters.

29

No significant differences were found.
The sums of x2 were 3 07 in Group IV
and 8-07 in Group V: with 12 d.f. they are
devoid of significance.

Histologically the thymic tumours
were poorly differentiated lymphosarco-
mata.

Tumours other than thymic lymphomata
among survivors at 39 weeks

Figure 3 compares the cumulative
probability of dying with a tumour at
any site (excluding mice with thymic
lymphomata) among animals surviving
at least 39 weeks after treatment in
groups receiving 50 ,ug/g NMU at 1, 21
or 70 days of age (Groups IV, V and VI).
From 50 weeks after treatment the cumu-
lative probability of dying with tumours
was somethat higher in Group IV (treated
at 1 day of age). On the other hand, no
differences appear to exist between mice
treated at 21 and 70 days. The average

0.7 -

co

E   0.6_
0
0.

E

-% 0.53

.2
-ct

CO  0.4_

0..

o   0.2

Q
co

.0 0.1
0

L.

I~~~~~~~~~~~~~~~~~~~~~~~~~~~~~~~

433

B. TERRACINI, M. C. TESTA, J. R. CABRAL AND L. ROSSI

E
0

0.9-

CD)

E   0.87
0)

.E 0.4 .

-o

0)  0.6.

U)

C0.5_

CO

0   0.4

0

sx  0.3_

._

0) 0.2_
~00.

-

._

QL

+   O S

0

0
0

0a
a

00.0

S

* O

a

0

O

0
0
0

O 0

0.0

0.
OS

* 0:

0

0
0o

0

o

O

0

0

0

00

1  0                         00
00            ~~~0

I        I     I           I

19  19 3 9   6 9   7 9   8 9   9 9   3 9 1X9

39   49    59    69    79   89     99   39   119

Weeks since treatment

Fio.:. 3. Probability of (lying with a tumour at arny site (exclufding thymic lymphomata) from the

40th week after a sinigle injectioni of 50 ylg/g NMU.

O Controls (Groulp I)

A Mice treated at 1 day (Group IV)

AlMice treated at 21 days (Group V)

O AMice treated at 70 (lays (Group VrI)

number of tumours at sites other than
the thymus in mice dying between 40 and
73 weeks after treatment (time of death
of the last mouse of group IV) was 1P9
per mouse in Group IV, 2 5 in Group V
and 2A4 in Group VI. It does not seem
that the higher probability of dying with
tumours shown by mice of Group IV
reflects a broader spectrum of tumour
induction when the treatment was given
early in life.

Dose-related differences in cumulative
probabilities of dying with tumours at
any age are far more obvious (Fig. 4).
For both doses of 50 and 25 ,ug/g NMU
and regardless of the age at treatment,
the end point is above 0 9. However,
for treatment at either 1 or 70 days

there is a consistent difference between
the effects of 50 and 25 pg/g, since the
two curves are separated by a distance
corresponding to 15-25 weeks. A plaus-
ible explanation is that the higher dose
induced more tumours per animal than
the lower one. Between 40 and 73 weeks
of age (time of death of the last mouse in
Group IV) the average number of tumours
per dying animal was 1-1 in Group II
and 1-9 in Group IV. Similarly, between
40 and 100 weeks of age (time of death
of the last mouse in Group VI) the
average number of tumours per dying
mouse was 1 3 in Group III and 2.3 in
Group VI.

Treatment with 5 pg/g NMU either
at 1 day or at 70 days of age did not

434

AGE AND DOSE IN CARCINOGENESIS BY NMU

0

o0

0

C

I     I    I     I    I     I    I     I

39   49   59    69   79    89    99  109   119

00

0

00

0   0

O CY
?     0

8 0

a

0

oP   0

0

0 o

o '0

0(9LJ 04

04?

c00

I    I    I   I

39  49  59  69  79  89  99  109  119

Age (weeks)

Fic. 4.-Probability of (lying with a tunio>ir at any site (excluding thymie

the 40th wveekz of ti(ge in C3Hf imlice given a single inljectionl of X-MU.

OControls (Gr ouip I)

v25 iyg/g at 1 day (Grotip II)

o 25 ,og/g at 70 (lays (Group Ill)
*50 pg/g at I day (Gr(olip 1 V)

o 50 1g/g at 70 (lays (Group V')

produce an obvious increase over the
controls in the probability of dying with
a tumour at any site.

Tumours of the forestomach

Of 87 mice with tumours of the
forestomach, 42 had at least one carcinoma
of the forestomach and the other 45
had one or more papillomata (Table II).
A sizeable incidence of carcinomata of
the forestomach was found in Groups V
and VI, receiving 50 ,tg/g NMU at 21
and 70 days of age respectively. Fig. 5
represents the distribution of the cumula-
tive probability of dying with a carcinoma
of the forestomach in these two groups:
when time since treatment is considered,
the two lines are almost overlapping,
indicating that the two treatments were
equally effective. Average time elapsing
between treatment and death with car-

a,

E
0
C

._

...

m

Co
t-l

._

.r-0

0
0
o6

1.Q-
0.9
0.8_
0.7

0.6

0.5
0.4

0.2.

0.1.

I 11-1l )holIn tfl)  l)eVOTt(l,

0
0

0
0

0
0

*  0

0

0 0
* 0

I  I  I  I  I  I  I  I  I

10   20   30   40   50   60    70   80   90   100

Age (weeks)

FIC. 5. Probability of (lying with a carcini-

oma of the forestomach in C3Hf mice given
a single injection of 50,ug/g NMU.

*Treate(d at 21 (lays (Gr-ouip V)

O'Treated at 70 (lays (Group - VI)

435

1 .0
*; 0.9..

: E 0.7_
0 0

E Qm o.6_

.H ._

*i?. E

0.

0.4-V

=5:0.3_

O 0.2-

, 0.1_
O.

31

T,,ff,

.0

4

4t

I        .4L

-?L
4L
4L

0%

3

B. TERRACINI, M. C. TESTA, J. R. CABRAI, AND L. ROSSI

TABLE IV. Incidence of Lung Adenomata, Liver-cell Turmours, Ovarian Tunmours,

Uterine and Vaginal Turnours and Orbital Gland Tuimours in C3Hf/Dp Mice Receiving

a Single Injection of NVM U Referred to Survivors at 40 Weeks of Age

AMice (lying
with luing
acleinomata
No.      0

6      24
4      12

1:1     72l-+

7      54++
8      40

12      60++

8      80++
4     1O0++
14      74++
21      68++
23      62++
18      78++

Mlice (lying
with livei

cell tumours
Noo.     0/o

1       4
13      38

2      11

9      69*
2      10
12      60

,3     30
4     100*
1       5

21      68+

2       5
8      35

AMice dying
with ovarian

tumours

3       2%

3      12

Mice (lying   Mice (lying
with uterine  with tumours
or vaginal      of the

ttumouirs   orbital glandls

,_I __  - -    f          A

No.

1

O !

0

4

2      11        1       6

4      20       6       30+

0       0      0
9      47+t     1
16      43+t    7

0
5
19

+ Difference vs controls l' < 0  i05.
++ Difference vs cointiols P < 0 01.

* If SS of Grouips II ain(l IV are considlered together P < 0 05.
t If 44 of Groups V and(l VI are consideredl together P < 0 01.

cinoma of the forestomach was 66-0 ? 6 1
weeks among the 6 mice of Group II,
60*7 ? 4-5 weeks in the 15 mice of Group
V and 656 + 44 weeks in the 17 mice of
Group VI.

In Group III, of 40 mice receiving
25 ,ug/g NMU at 70 days of age, 2 died
with a carcinoma (respectively 46 and
64 weeks after treatment), and 8 had one
or multiple papillomata of the fore-
stomach. The overall incidence of 10/40
mice developing stomach tumours was
higher than that of 5/59 found in the
controls (P < 0.05). Among mice re-
ceiving 5 ,tg/g NMU at 1 or 70 days of
age the incidence of stomach papillomata
was, if anything, lower than in the
controls.

Other tumours

Target organs of the carcinogen NMU
included lungs, liver, kidney, intestine,
ovary, uterus and vagina (Table II).
The incidences of some of these tumours
among survivors at 40 weeks of age are
reported in Table IV.

A significant increase (P < 0.01) of
the proportion of mice dying with lung
adenomata was found in both sexes in

No.

0
1
0
0
1

0
0
2
6
1
3

groups given 50 ,ag/g NMU at any age
or 25 jag/g at 1 day as well as in males
receiving 25 ,tg/g at 70 days.

Liver cell tumours were increased
over the controls (P < 0 05) in males
receiving 50 ,tg/g at 21 days as well as
in those treated with either 50 or 25 /tg/g
at 1 day if considered together. No
evidence of hepatocarcinogenicity was
found in any of the other treated animals.

Renal nodules ranging between 0'2
and 1-0 cm in diameter were observed in
21 mice of either sex receiving 50 ,ug/g
NMU either at 1, 21 or 70 days and in
none of the other groups. Histologically,
they were benign solid or papillary
adenomata, with the exception of one
sarcoma and one very atypical epithelial
tumour. Mice exposed at 70 days ap-
peared more susceptible than those treated
at 21 days, but the difference is of
borderline significance.

A total of 37 females died with an
ovarian tumour: a few of these were
complicated by peritoneal haemorrhage
which might have caused death. Histo-
logically, they were of the granulosa-cell
type with the exception of one luteoma
and one tumour too decomposed for a
diagnosis.   A    significant  increase

Group

I
II
III
IV
V
VI

Survivors

at

40 weeks

4 25
3 34
4 18
,@ 1.3
4 20
J; 20
X 10
c3 4
4 19
d; 31
4 37
,; 23

o3
,lo

.3

11
19

3
1 3

436

AGE AND DOSE IN CARCINOGENESIS BY NMU

(P < 0.05) over the controls was observed
among mice given 50 ,ig/g NMU at 21 or
70 days: incidences were 9/19 and 16/37
respectively vs. 3/25 ill the controls.
No ovarian tumours were found in the
10 females treated with the same dose
at 1 day and surviving 40 or more weeks.

Twenty-one females at death had a
fibroma or fibrosarcoma in the uterus or
in the vagina. Only in mice treated with
25 or 50 ltg/g at 70 days was there an
increase of marginal significance in the
incidence of these tumours.

Orbital gland tumours were increased
over the controls in males receiving 50
,ug/g NMU at 21 or 70 days but not in
other groups.

DISCUSSION

In terms of target organs and effective
carcinogenic doses of a single administra-
tion of NMU to mice, the present in-
vestigation is confirmatory of previous
studies on other strains of mice (Graffi
and Hoffmann, 1966; Terracini and
Stramignoni, 1967; Kelly et al., 1968;
Frei, 1970; Joshi and Frei, 1970; Terracini
and Testa, 1970; Frei, 1971). The spor-
adic occurrence of neurogenic tumours
in the present series as well as in previous
studies differs from findings following a
single administration of the related com-
pound N-nitroso-N-ethylurea (Searle and
Jones, 1972; Vesselinovitch et al., 1974).

Reviews on the role of age at treat-
ment in chemical carcinogenesis lead to
the conclusion that neonates are more
susceptible than older animals to the
induction of lymphomata, liver-cell tu-
mours and lung adenomata, whereas
results for other target organs are less
definite (Toth, 1968; Della Porta and
Terracini, 1969). The particular suscepti-
bility of some organs of newborn mice
to chemical carcinogenesis has been at-
tributed to a series of host-related factors
such as metabolic maturation, immuno-
logical or hormonal conditions, different
functional status of the target organs,
etc. (Toth, 1968; Della Porta and Terracini,
1969).

The progressive decrease of the suscept-
ibility to thymic lymphoma induction
from birth to 10 weeks of age confirms
previous studies with NMU itself (Terra-
cini and Testa, 1970), urethane (De
Benedictis et al., 1964; Vesselinovitch,
Mihailovich and Itze, 1970), DMBA (Toth,
Rappaport and Shubik, 1961) and N-
nitroquinoline- 1-oxide (Nishizuka, Naka-
kuki and Sakakura, 1964). In one of
such studies, (C57BL x C3H)F1 mice
aged 25 weeks were found completely
resistant to treatment with urethane at
a dose which was highly active in newborn
mice (Vesselinovitch et al., 1970).

On the other hand, when a com-
parison is made between groups of the
carcinogenic action on all organs excluding
the thymus, age at treatment seemed to
play a minor role. Analyses of tumour
incidences in individual organs is limited
by interference caused by intercurrent
deaths. However, hepatocarcinogenicity
was confined to male mice receiving 25
or 50 ,ug/g NMU at 1 or 21 days of age.
In a previous study on the same colony
of C3Hf/Dp mice, a single administration
of 1 mg urethane at birth was equally
effective on both sexes (Della Porta et
al., 1967).

The susceptibility to carcinogenesis
of the ovary and orbital glands seems
to be lower in newborns than in animals
aged 21 or 70 days, in confirmation of
previous studies with urethane and N-
nitroso-N-ethylurea (Vesselinovitch and
Mihailovich, 1967; Vesselinovitch et al.,
1974).

In the present series, the probability
of dying with a carcinoma of the fore-
stomach following 50 ,tg/g was the same
in mice treated at 21 days and in those
treated at 70 days of age. Among ani-
mals given 25 ,tg/g NMU, the proportion
developing benign or malignant tumours
of the forestomach was 12/41 in those
treated at birth and 10/40 in those
treated at 70 days of age. Although 6
of the former had a carcinoma vs. 2 of
the latter, these differences are hardly
significant. In a previous study with

437

438         B. TERRACINI, M. C. TESTA, J. R. CA1BRAL AND L. ROSSI

N-nitroso-N-ethylurea, mice treated at
birth were slightly less susceptible to
stomach carcinogenesis than those treated
at 15 or 42 days of age (Vesselinovitch et
al., 1974).

Lifetime analysis in the present series
indicates that lymphomata following ex-
posure to 50 ,ug/g NMU at birth not only
are more frequent but also progress to
death more rapidly than those induced
later in life. By and large this is con-
firmatory of previous investigations with
NWU (Terracini and Testa, 1970), DMBA
(Toth et al., 1961) and urethane (De
Benedictis et al., 1964) as well as with
Gross' passage A virus (Axelrad and Van
der Gaag, 1962).

The difference in the mechanism of
carcinogenesis reflected in this result
might take place either at the time of
the interaction between carcinogen and
cell components or during tumour de-
velopment. It is unlikely that age at
treatment affects the persistence in the
target organ of NMU, as this is an un-
stable compound, rapidly broken down
without enzymic activation (Druckrey et
al., 1967; Swann, 1968). Morphological
differences of the thymus between new-
born and adult mice (Axelrad and Van
der Gaag, 1962) do not appear to be
involved, since thymic lymphomata in-
duced in newborn mice with low doses
of NMU progressed relatively slowly.
In addition, no morphological differences
were found between lymphomata occur-
ring in mice treated at birth and those
found in mice treated later in life.

The different rate of progression of
thymic lymphomata related to age at
treatment more likely reflects differences
in tumour-host relationships during the
early phases of tumour development.
" Natural " age-related differences such
as immunosurveillance and the hormonal
situation do not seem to be relevant since
thymic lymphomata induced by a low
dose of NMU at birth progressed as
slowly as those induced by a high dose
given at 10 weeks of age.

On the other hand, an age-related

systemic effect of NMU on the general
status of the animals is reflected in the
degree of body growth inhibition related
to age at treatment. A role of NMU-
induced immuno-depression is possible,
but the impact of age at treatment on
this effect has not been investigated
(Parmiani, Colnaghi and Della Porta,
1971). This hypothesis is compatible
with the theory that chemical induction
of lymphomata in mice is mediated by
viral activation (Kaplan, 1967).

Age-related factor(s) affecting tumour-
host relationship do not appear to be
involved in the mechanism of carcino-
genesis for the stomach, where progression
was independent of age at treatment.
This discrepancy between thymic lympho-
mata and carcinomata of the forestomach
is reminiscent of a previous observation
that the immunological status of NMU-
treated rats does not affect equally the
induction of tumours of the nervous
system and that of tumours of the
bladder (Denlinger et al., 1973). This
again suggests that host-tumour inter-
action may be quite different according
to the organs involved.

The authors are grateful to Dr G.
Della Porta for helpful discussions and
to Mr R. Rubertelli and Ms G. Pasquini
for their technical help.

REFERENCES

AXELRAI), A. A. & VAN DER GAAG, H. (1962)

Susceptibility to Lymphoma Induction by Gross'
Passage A Virus in C3Hf/Bi Mice of Different
Ages: Relation to Thymic Cell Multiplication
and Differentiation. J. natn. Cancer Inst., 28,
1065.

DE BENEDICTIS, G., CHIECO-BIANCHI, L., TRIDENTE,

G. & FIORE-DONATI, L. (1964) Infltienza del
Fattore Eta sullo Sviluppo di Linfomi Maligni
e di Epatomi in Topi Swiss Trattati con Uretano.
Boll. Soc. ital. Biol. sper., 40, 610.

DELLA PORTA, G., CAPITANO, J., PARMI, L. &

COLNAaHI, M. I. (1967) Cancerogenesi da Uretanio
in Topi Neonati, Lattanti e Adulti dei Ceppi
C57BL, C3H, BC3FI, C3Hf e SWR. Tunmori, 53,
81.

DELLA PORTA, G. & TERRACINI, B. (1969) Chemical

Carcinogenesis in Infant Animals. Progr. exp.
Tumnor Res., 11, 334.

DENLINGER, R. H., SWENBERG, J. A., KOESTNER, A.

& WECHSLER, W. (1973) Differential Effect of

AGE AND DOSE IN CARCINOGENESIS BY NMU         439

Immunosuppression on the Induction of Nervous
System and Bladder Tumors by N-Methyl-N-
Nitrosourea. J. natn. Cancer Inst., 50, 87.

DRUCKREY, H., PREUSSMANN, R., IVANKOVIC, S.

& SCHMXHL, D. (1967) Organotrope Carcinogene
Wirkungen bei 65 Verschiedenen N-Nitroso-
Verbindungen an BD-Ratten. Z. Kreb8forsch.,
69, 103.

DRUCKREY, H., STEINHOFF, D., PREUSSMANN, R.

& IVANKOVIC, S. (1964) Erzeugung von Krebs
durch einie einmalige Dosis von Methylnitroso-
harnstoff und verschiedenen Dialkylnitrosaminen
an Ratten. Z. Krebsforsch., 66, 1.

FREI, J. V. (1970) Toxicity, Tissue Changes and

Tumor Induction in Inbred Swiss Mice by
Methylnitrosamine and -amide Compounds. Can-
cer Res., 30, 11.

FREI, J. V. (1971) Tumour Induction by Low

Molecular Weight Alkylating Agents. Chem.
biol. Inter., 3, 117.

GRAFFI, A. & HOFFMANN, F. (1966) Starke Leuk-

amogene Wirkung von N-Methyl-N-Nitroso-
harnstoff bei der Maus nach einmaliger Applika-
tion an neugeborene Tiere. Acta biol. med.
germ., 17, 33.

INTERNATIONAL AGENCY FOR RESEARCH ON CANCER

(1972) IARC Monographs on the Evaluation of
Carcinogenic Risk of Chemicals to Man. Vol. I.
Lyon: IARC.

JOSHI, V. V. & FREI, J. V. (1970) Effects of Dose

and Schedule of Methylnitrosurea on Incidence
of Malignant Lymphoma in Adult Female Mice.
J. natn. Cancer Imst., 45, 335.

KAPLAN, H. S. (1967) On the Natural History of

the Murine Leukemias (Presidential Address).
Cancer Res., 27, 1325.

KELLY, M. G., O'GARA, R. W., YANCEY, S. T. &

BOTKIN, C. (1968) Carcinogenicity of 1-methyl-l-
nitrosourea in Newborn Mice and Rats. J. natn.
Cancer Inst., 41, 619.

LEAVER, D. D., SWANN, P. F. & MAGEE, P. N.

(1969) The Induction of Tumours in the Rat
by a Single Oral Dose of N-Nitrosomethylurea.
Br. J. Cancer, 23, 177.

NISHIZUKA, Y., NAKAKUKI, K. & SAKAKURA, T.

(1964) Induction of Pulmonary Tumor and
Leukemia by a Single Injection of 4-Nitroquino-
line-l-oxide to Newborn and Infant Mice. Gann,
55, 495.

PARMIANI, G., COLNAGHI, M. I. & DELLA PORTA, G.

(1971) Immunodepression During Urethane and
N-Nitrosomethylurea Leukaemogenesis in Mice.
Br. J. Cancer, 25, 354.

SAFFIOTTI, U., MONTESANO, R., SELLAKUMAR,

A. R., CEFIS, F. & KAUFMAN, D. G. (1972)
Respiratory Tract Carcinogenesis in Hamsters
Induced by Different Numbers of Administrations
of Benzo(a)pyrene and Ferric Oxide. Cancer
Res., 32, 1073.

SEARLE, C. E. & JONES, D. L. (1972) Tumours

of the Nervous System in Mice Treated Neo-
natally  with N-ethyl-N-Nitrosourea.  Nature,
Lond., 240, 559.

SWANN, P. F. (1968) The Rate of Breakdown of

Methyl Methanesulphonate, Dimethyl Sulphate
and N-Methyl-N-Nitrosourea in the Rat. Bio-
chem. J., 110, 49.

TERRACINI, B. & STRAMIGNONI, A. (1967) Malignant

Lymphomas and Renal Changes in Swiss Mice
Given Nitrosomethylurea. Eur. J. Cancer, 3,
435.

TERRACINI, B. & TESTA, M. C. (1970) Carcinogenicity

of a Single Administration of N-Nitrosomethyl-
urea: A Comparison Between Newborn and
Five-week-old Mice and Rats. Br. J. Cancer,
24, 588.

TOTH, B. (1968) A Critical Review of Experiments

in Chemical Carcinogenesis Using Newborn
Animals. Cancer Res., 28, 727.

TOTH, B., RAPPAPORT, H. & SHUBIK, P. (1963)

Influence of Dose and Age on the Induction
of Malignant Lymphomas and Other Tumors
by 7,12-dimethylbenz(a)anthracene in Swiss Mice.
J. natn. Cancer Inst., 30, 723.

VESSELINOVITCH, S. D. & MIHAILOVICH, N. (1967)

The Neonatal and Infant Age Period as Biologic
Factors which Modify Multicarcinogenesis by
Urethane. Cancer Res., 27, 1422.

VESSELINOVITCH, S. D., MIHAILOVICH, N. & ITZE, L.

(1970) Comparative Studies on the Kinetics of
the Neoplastic Competence in Mice. Cancer
Res., 30, 2548.

VESSELINOVITCH, S. D., RAO, K. V. N., MIHAILO-

VICH, N., RIcE, J. M. & LOMBARD, L. S. (1974)
Development of Broad Spectrum of Tumors by
Ethylnitrosourea in Mice and the Modifying
Role of Age, Sex and Strain. Cancer Res., 34,
2530.

				


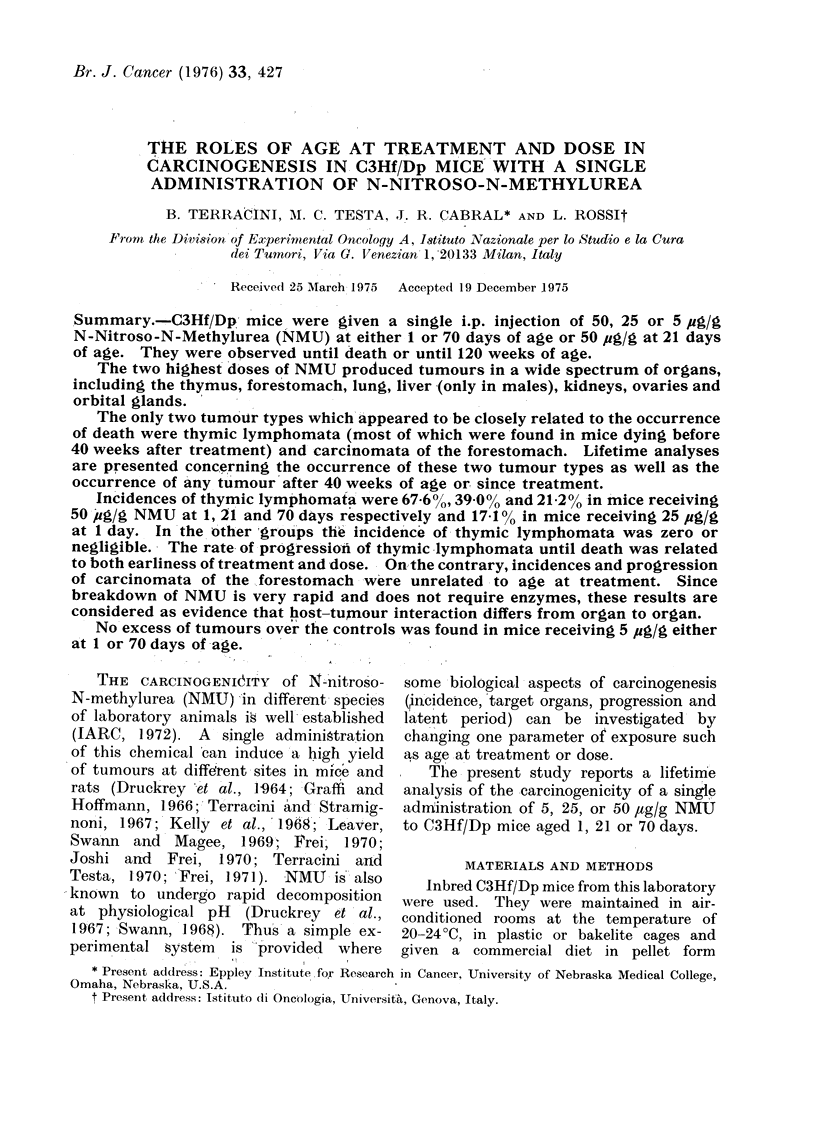

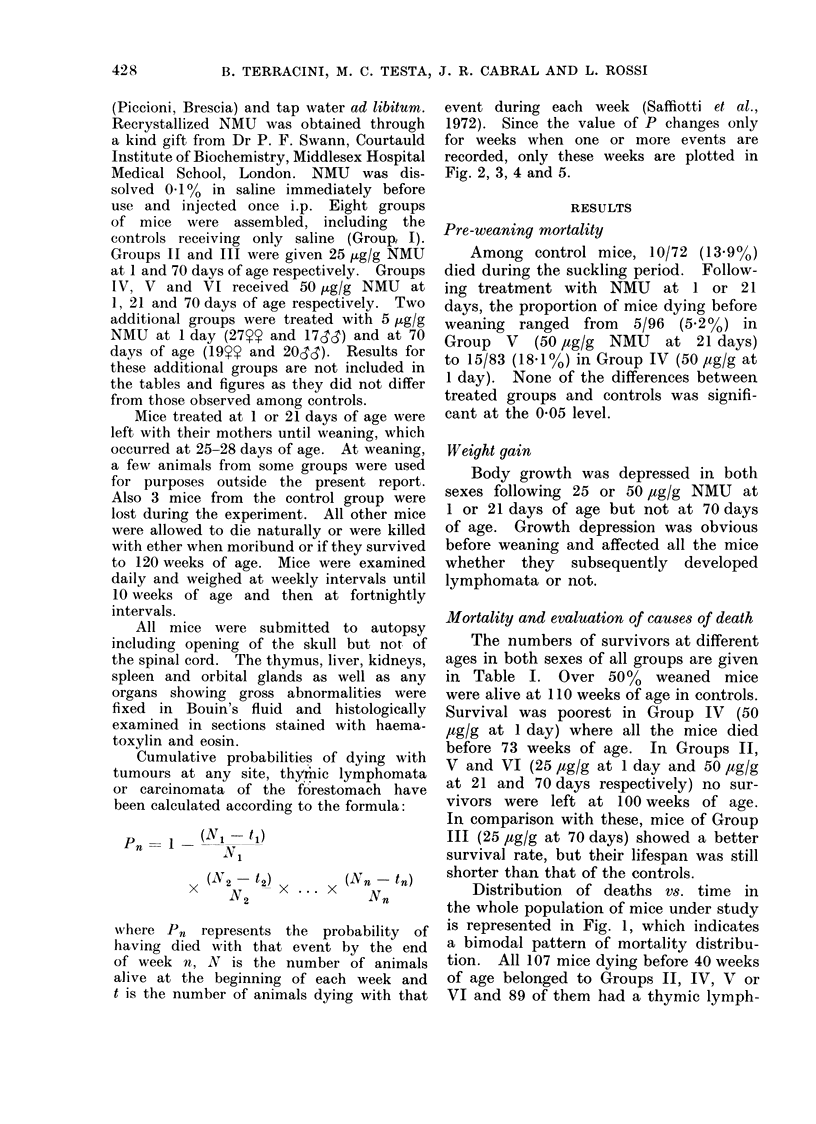

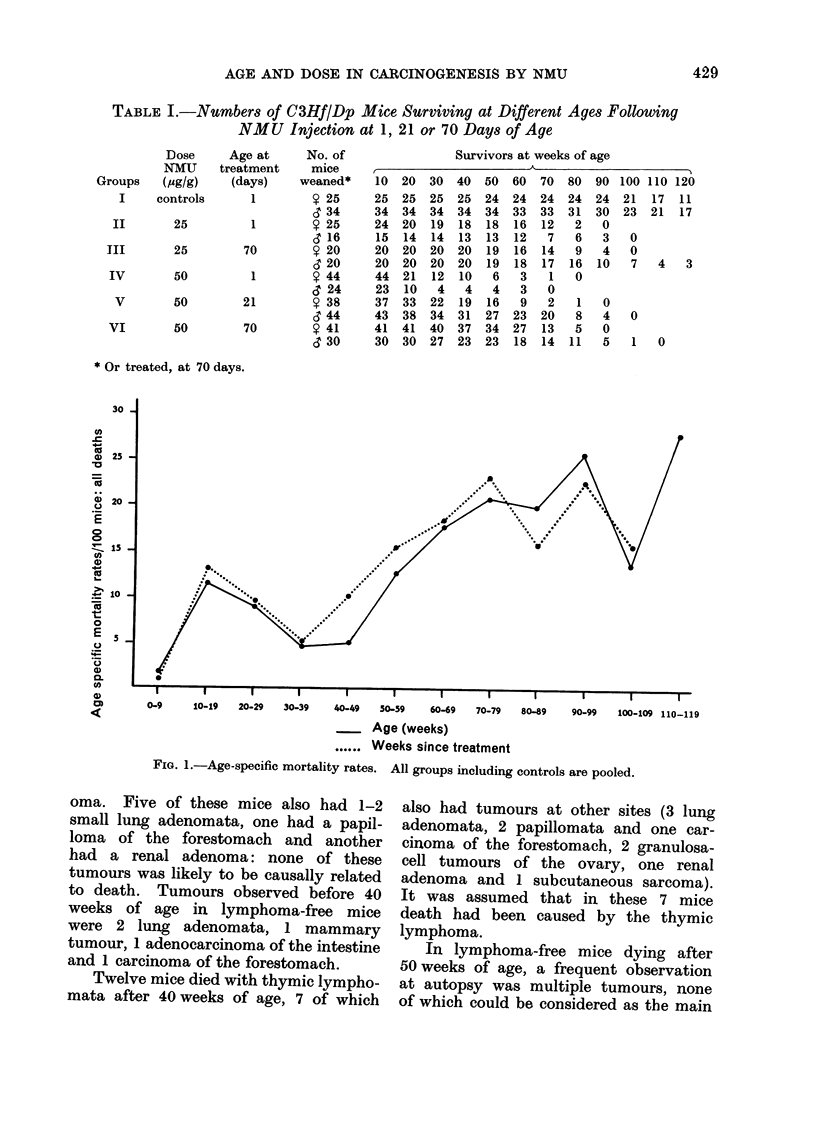

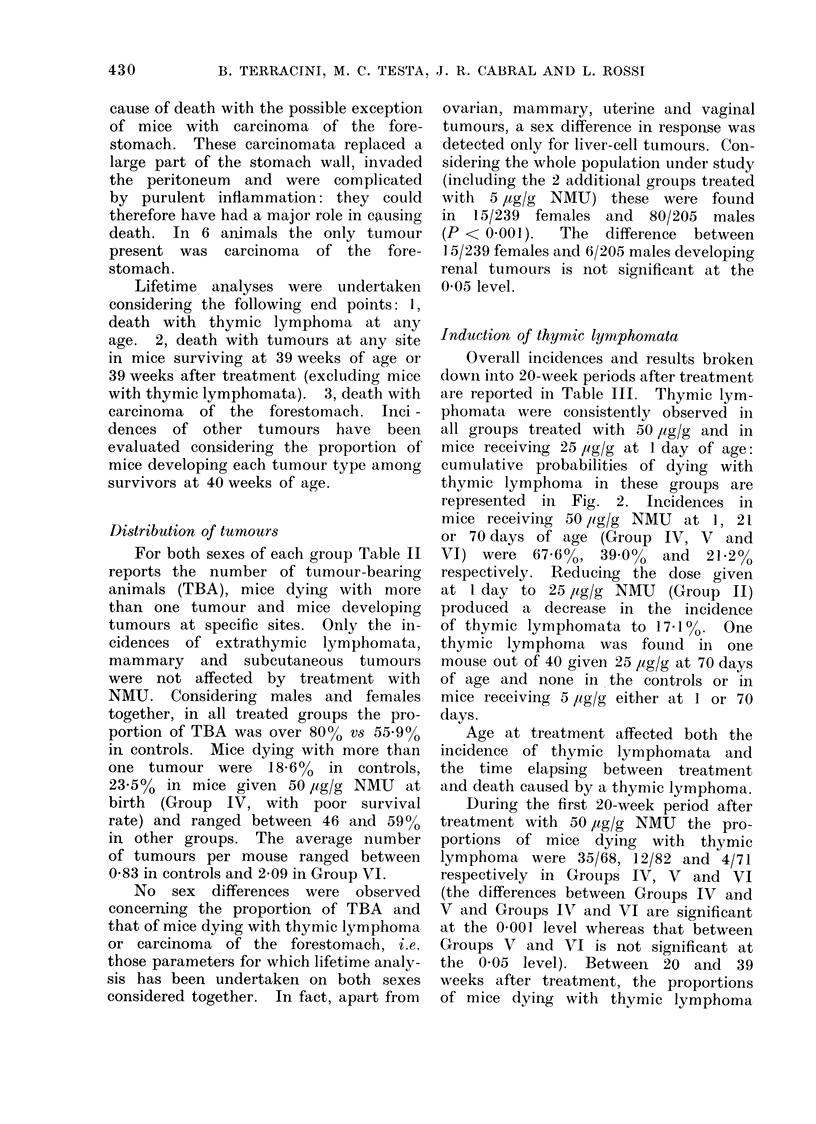

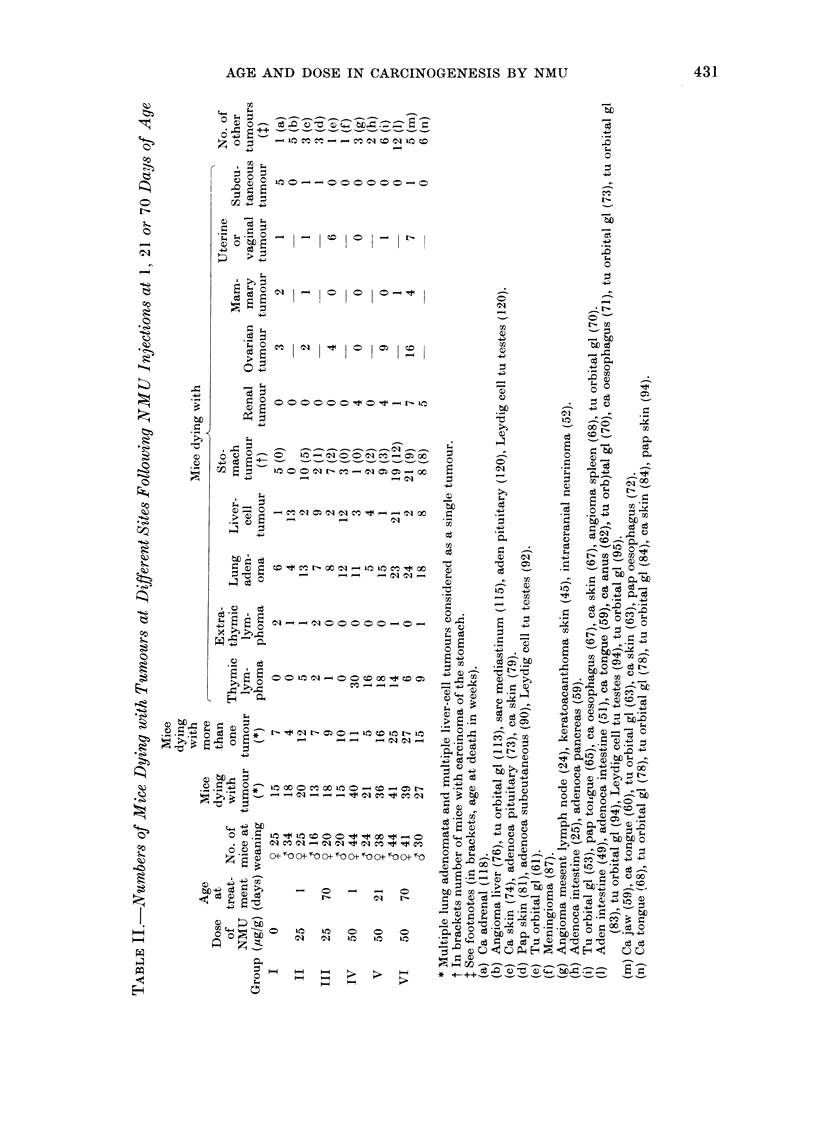

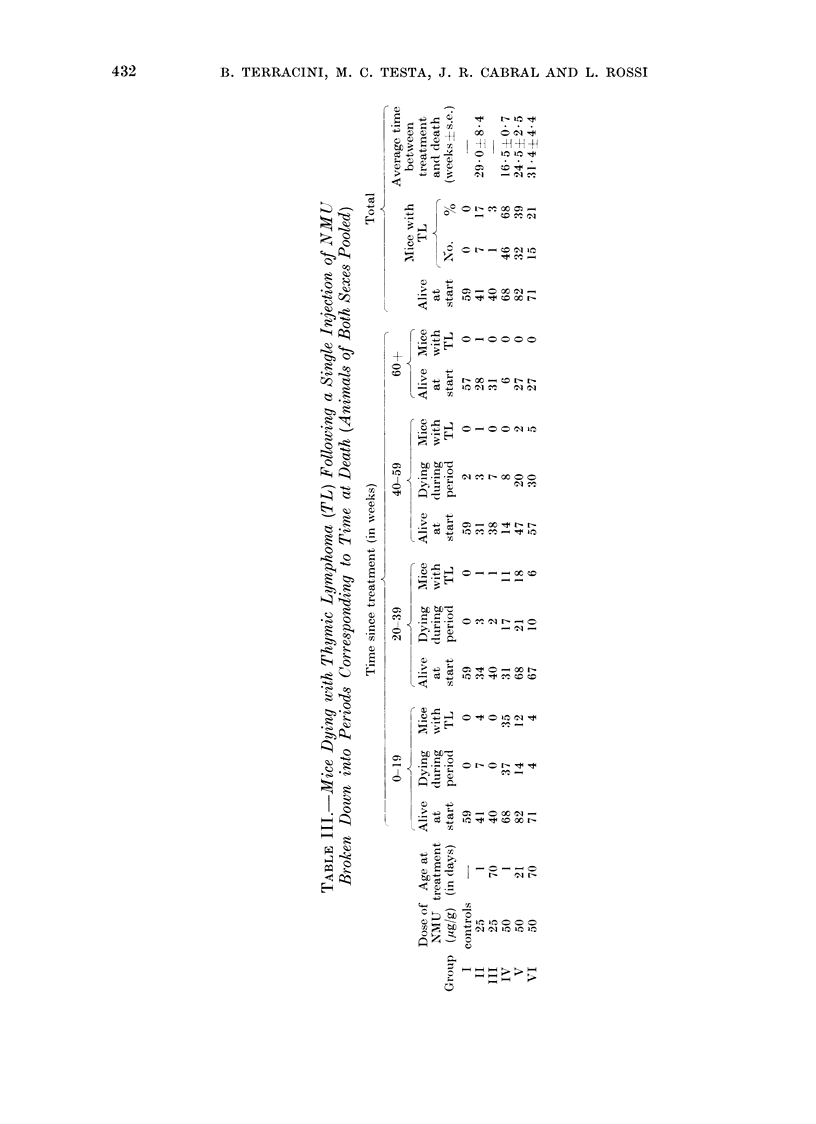

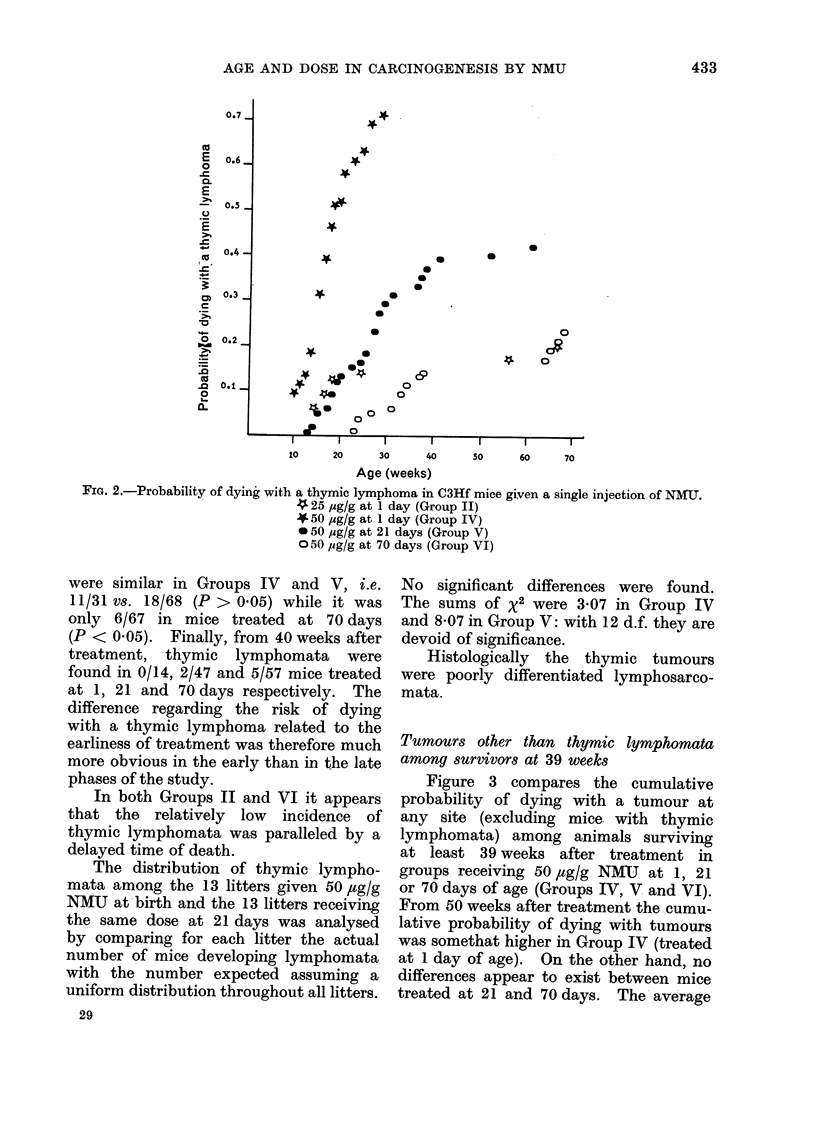

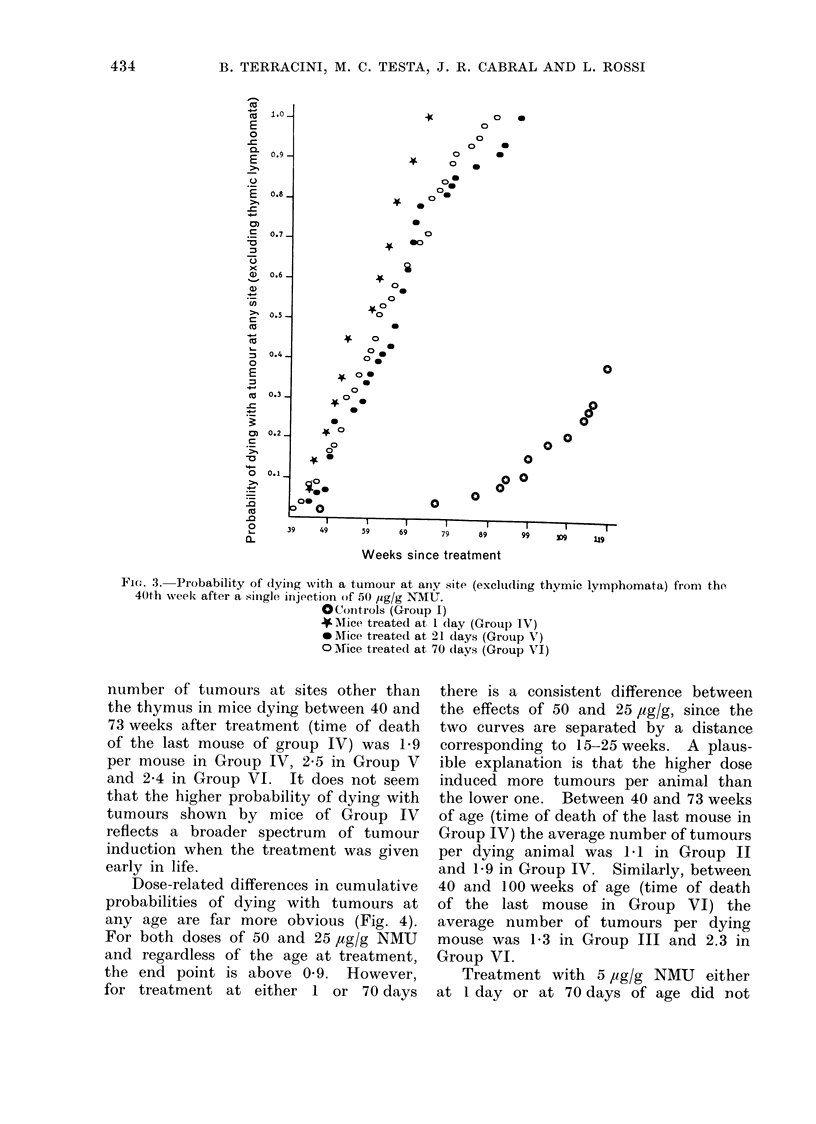

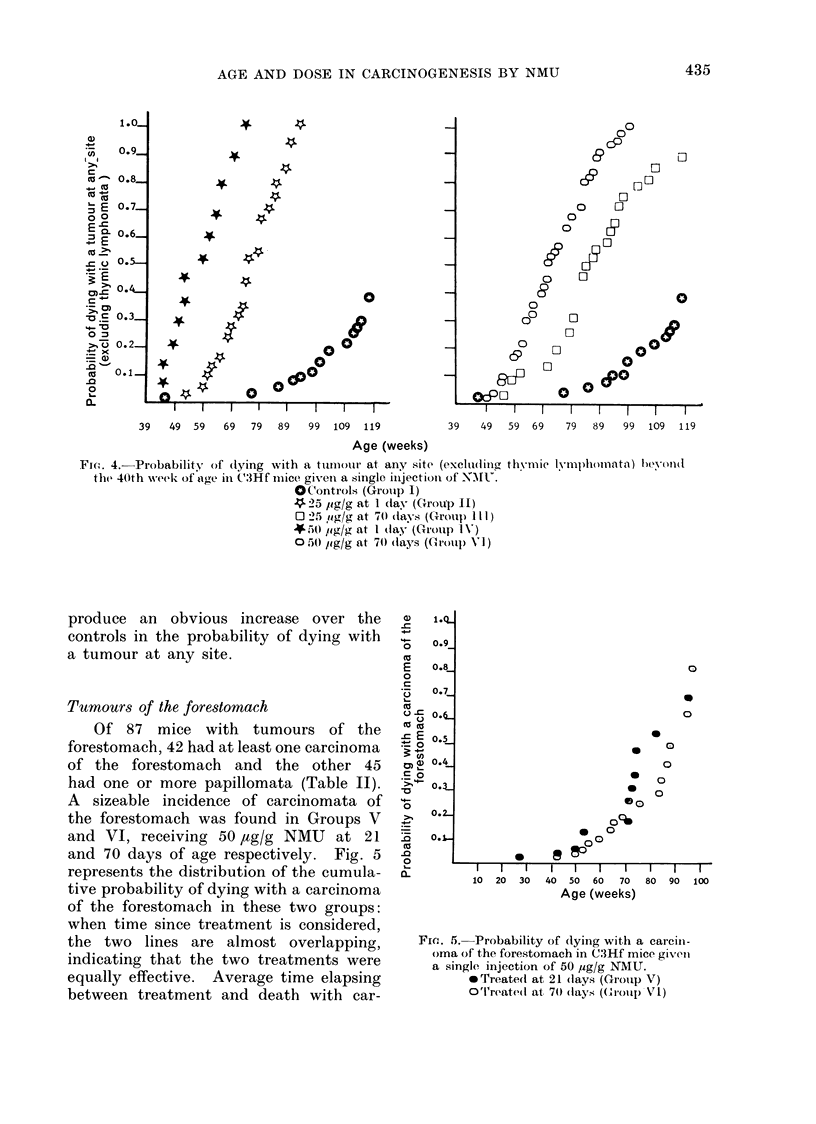

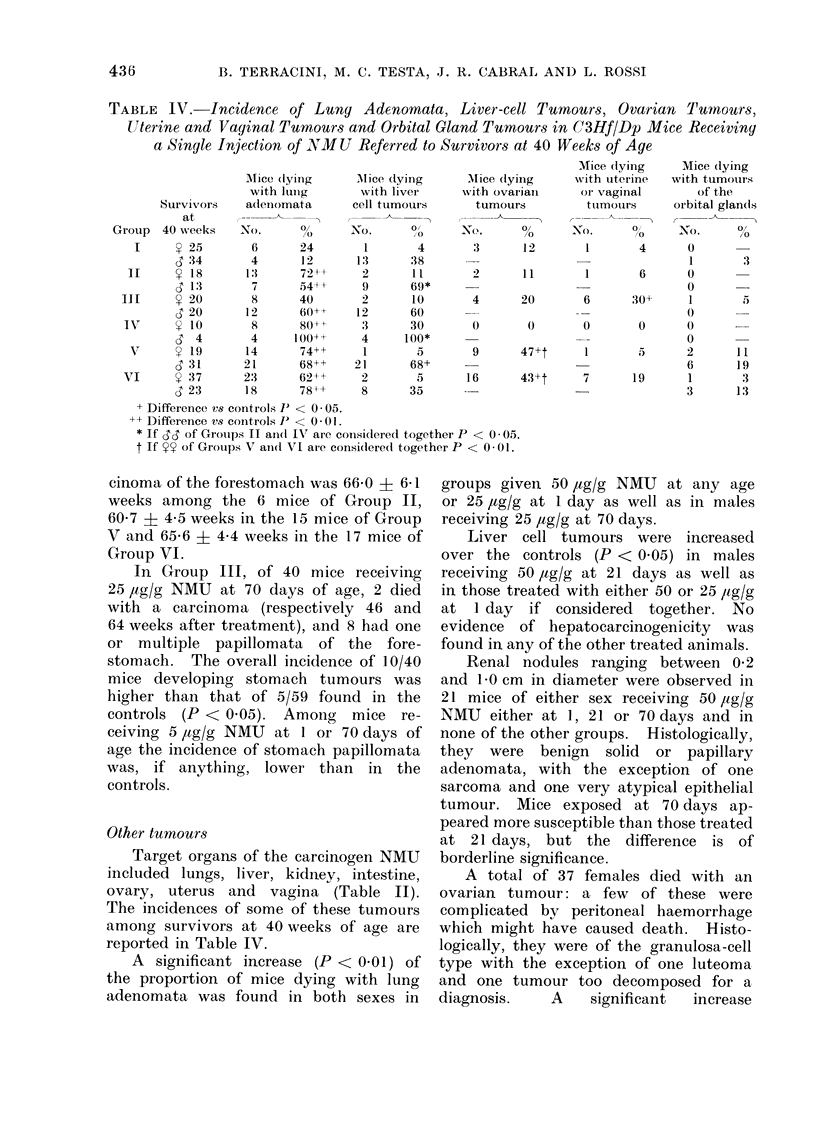

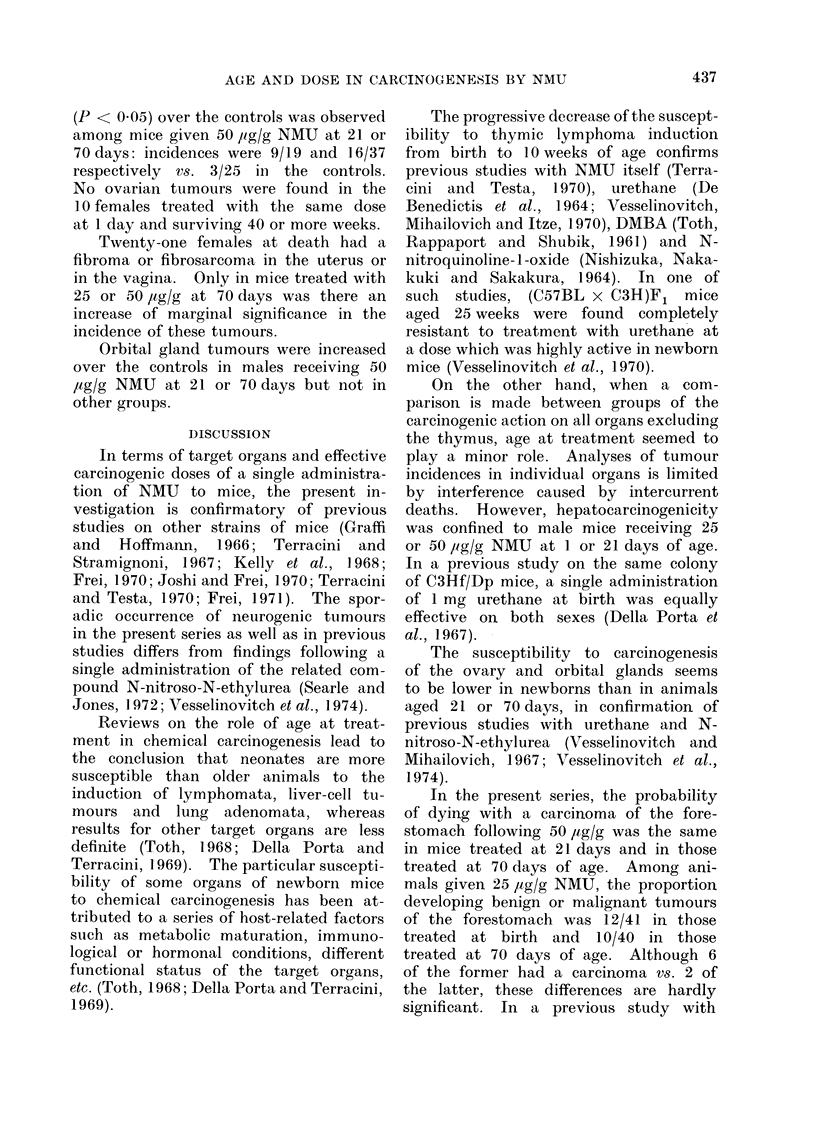

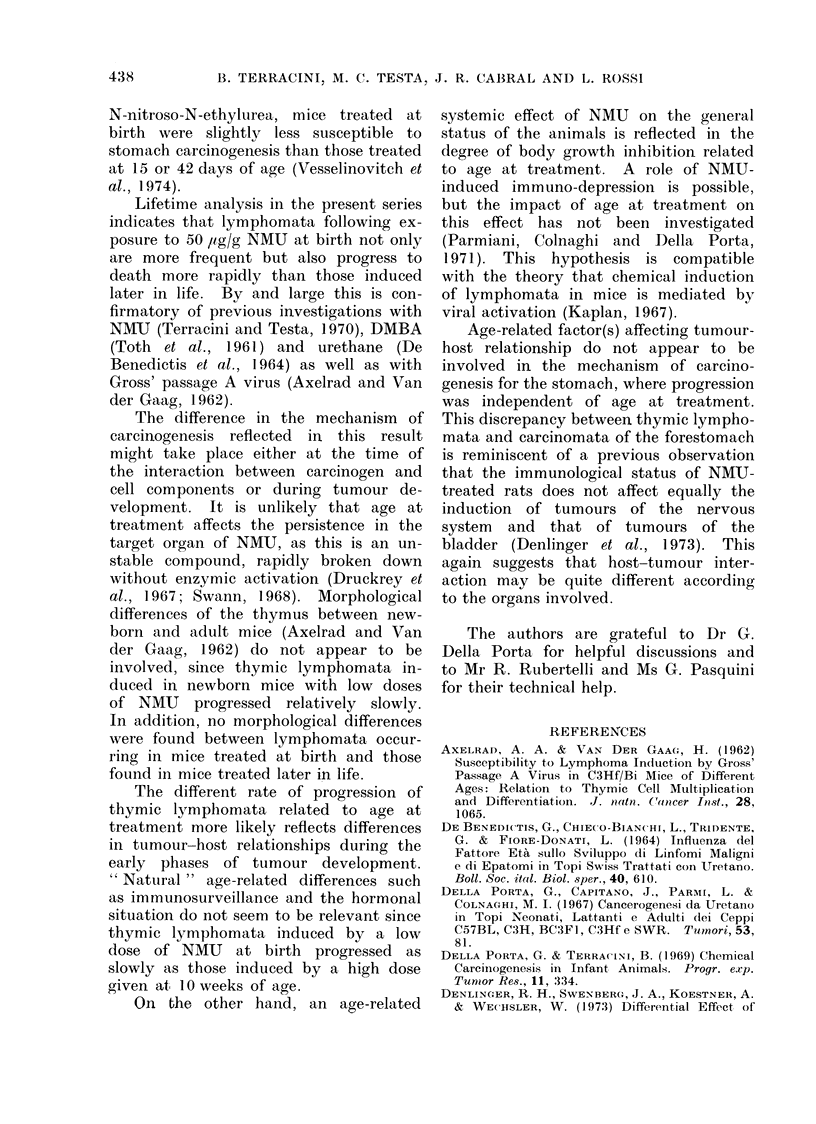

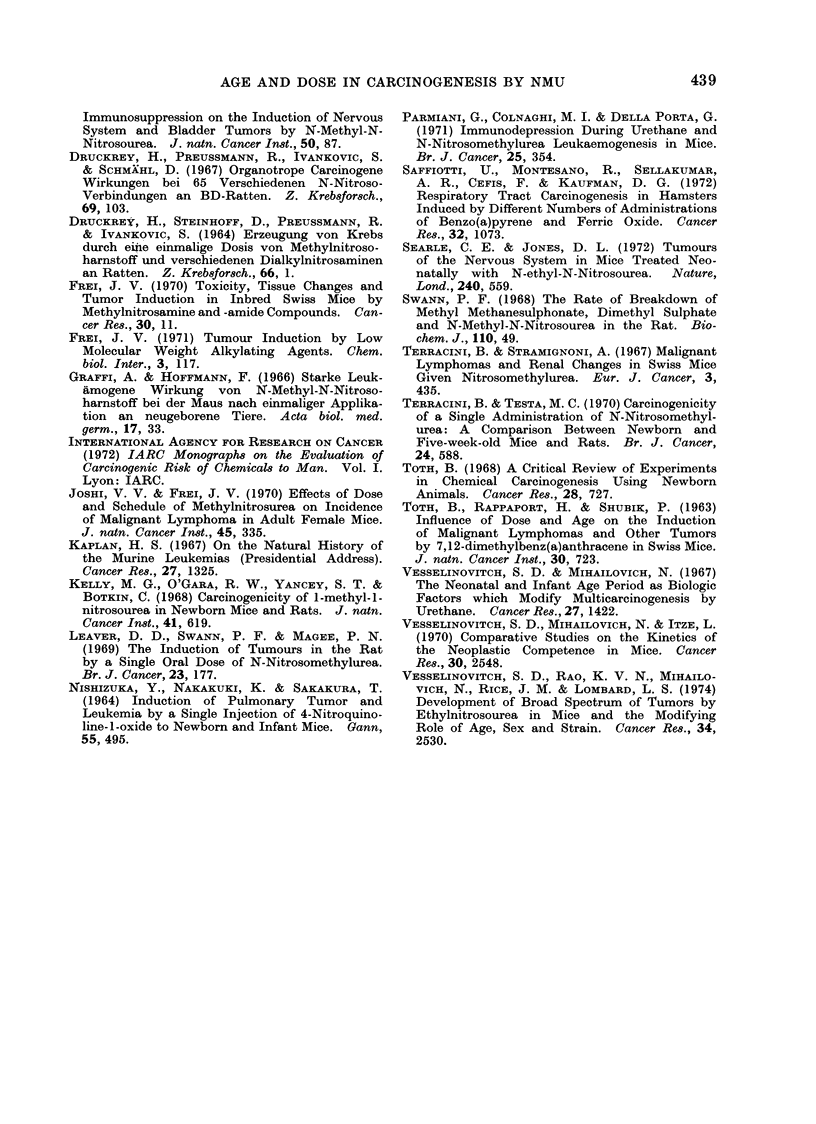

